# Supplementary material for: Phosphodiesterase Inhibition to Sensitize Non-Small-Cell Lung Cancer to Pemetrexed: A Double-Edged Strategy
Source: Cancers (Basel). 2024 Jul 6;16(13):2475. doi: 10.3390/cancers16132475 (PMC11240499; doi:10.3390/cancers16132475)
Supplement: Supplementary file 1 [file cancers-16-02475-s001.zip › cancers-2937367-supplementary.pdf]

Phosphodiesterase inhibition to sensitize non-small cell lung cancer to pemetrexed: a double-edge strategy

Anna V. Ivanina Foureau[1], David M. Foureau[2], Cody C. McHale[3], Fei Guo[2], Carol Farhangfar[1], and Kathryn F. Mileham[4].

[1] Translational Research, Levine Cancer Institute, Atrium Health, Charlotte, NC 28204, USA.

[2] Immune Monitoring Core Laboratory, Levine Cancer Institute, Atrium Health, Charlotte, NC 28204, USA.

[3] Molecular Targeted Therapeutics laboratory, Levine Cancer Institute, Atrium Health, Charlotte, NC 28204, USA.

[4] Thoracic Medical Oncology, Levine Cancer Institute, Atrium Health, Charlotte, NC 28204, USA.

Supplemental material

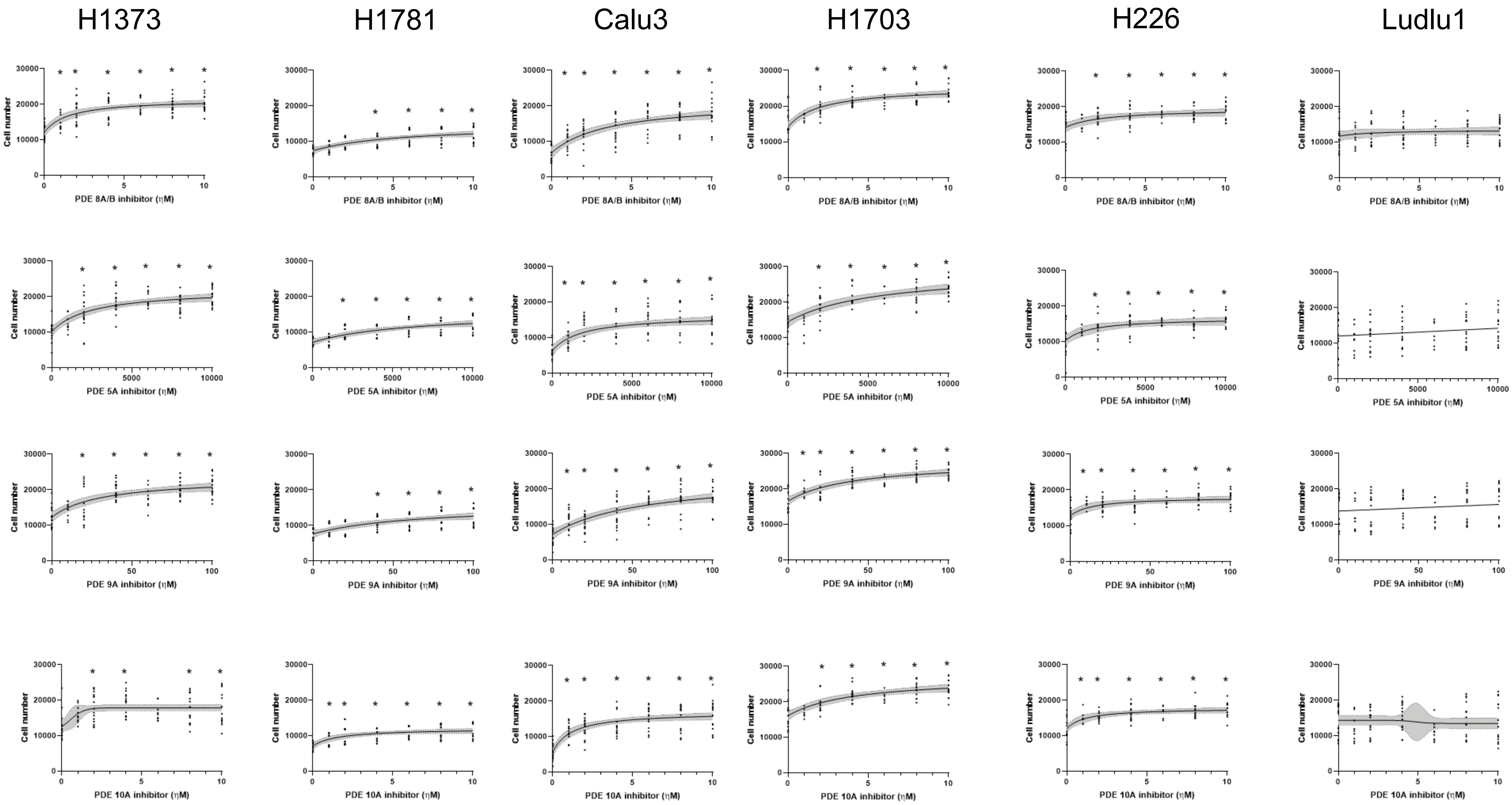

Supplemental Figure S1. PDEi stimulate NSCLC cell proliferation. NSCLC cell lines (H1373, H1781, Calu3, H1703, H226 and Ludlu1) in exponential phase of growth were exposed for 72h to PDE8i (0-6ηM), PDE5i (0-6000ηM), PDE9i (0-60ηM) or PDE10i (0-6ηM), followed by measurement of cell proliferation using WST-8 cell counting kit. Data presented as cell number, N=6-8. * Indicate significant differences from untreated respective control (P<0.05).

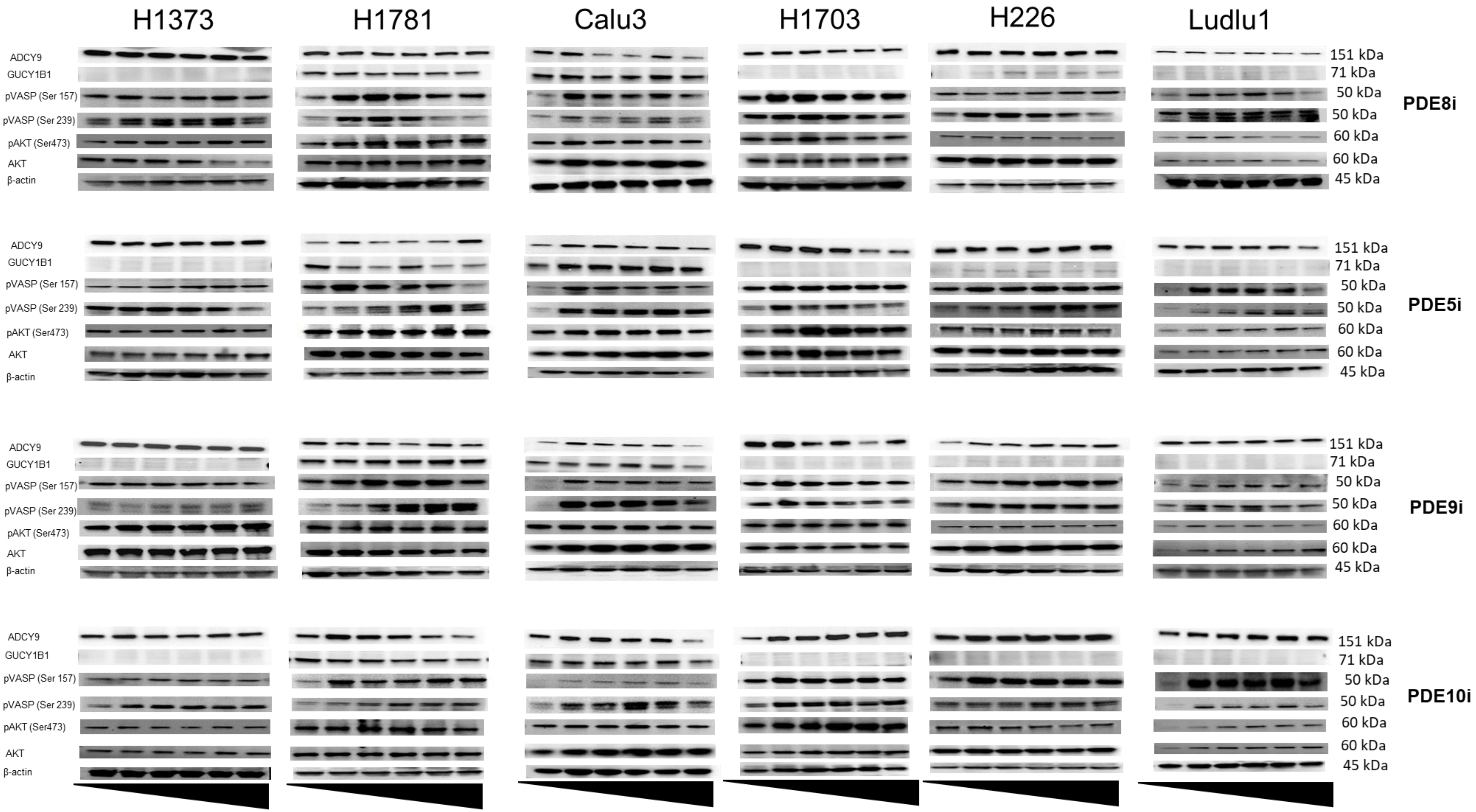

Supplemental Figure S2. PDEi effect on PKA/PKG signaling in NSCLC cell lines. NSCLC cell lines (H1373, H1781, Calu3, H1703, H226 and Ludlu1) in exponential phase of growth were exposed for 72h to PDE8i (0-6ηM), PDE5i (0-6000ηM), PDE9i (0-60ηM) or PDE10i (0-6ηM). Adenylate cyclase (ADCY) and soluble guanylate cyclase (GUCY1B1), indirect PKA and PKG activity measured by site-specific phosphorylation of vasodilator-stimulated phosphoprotein, pVASP157(PKA) and pVASP239(PKG)), phospho-AKT (pAKT (Ser473)) and AKT, determined by Western blotting. β-actin was used as the loading control.

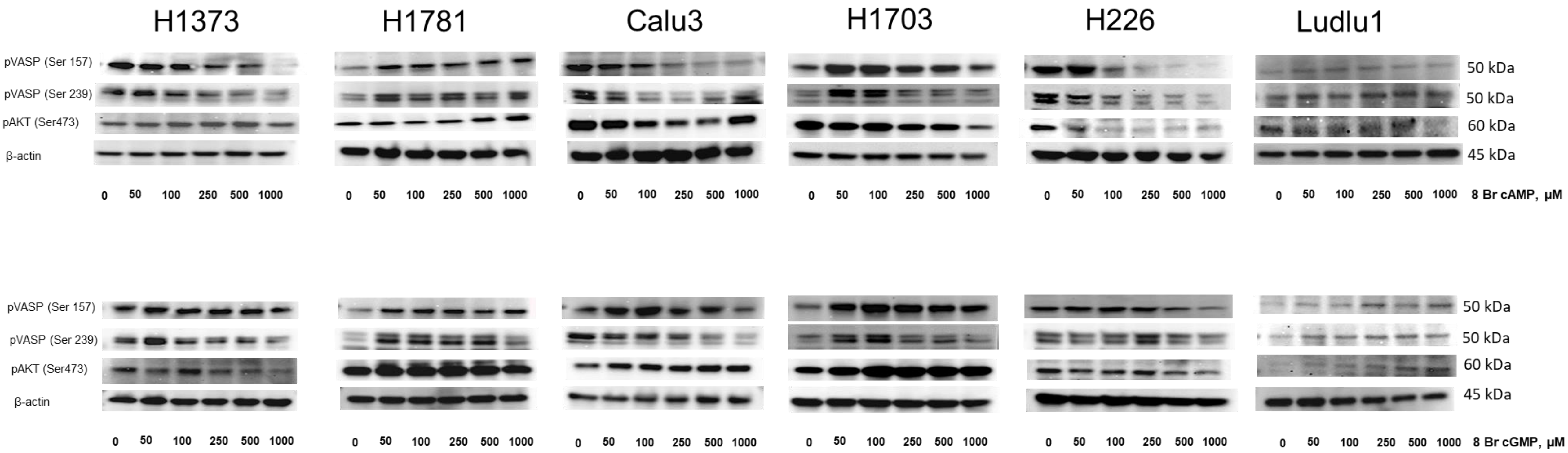

Supplemental Figure S3. Direct activators of PKA/PKG affect protein kinases A and G signaling in NSCLC cell lines. NSCLC cell lines (H1373, H1781, Calu3, H1703, H226 and Ludlu1) in exponential phase of growth were exposed to 8-Bromo-cAMP, sodium salt ,a PKA activator (0-1mM) or 8-Bromo-cGMP, sodium salt, a PKG activator (0-1mM) for 72h. Cell-specific activation of PKA and PKG by direct activators determined by phosphorylation of PKA target (pVASP157) and PKG target (pVASP239) and phospho-AKT (pAKT (Ser473)) measured by Western blotting. β-actin was used as the loading control.

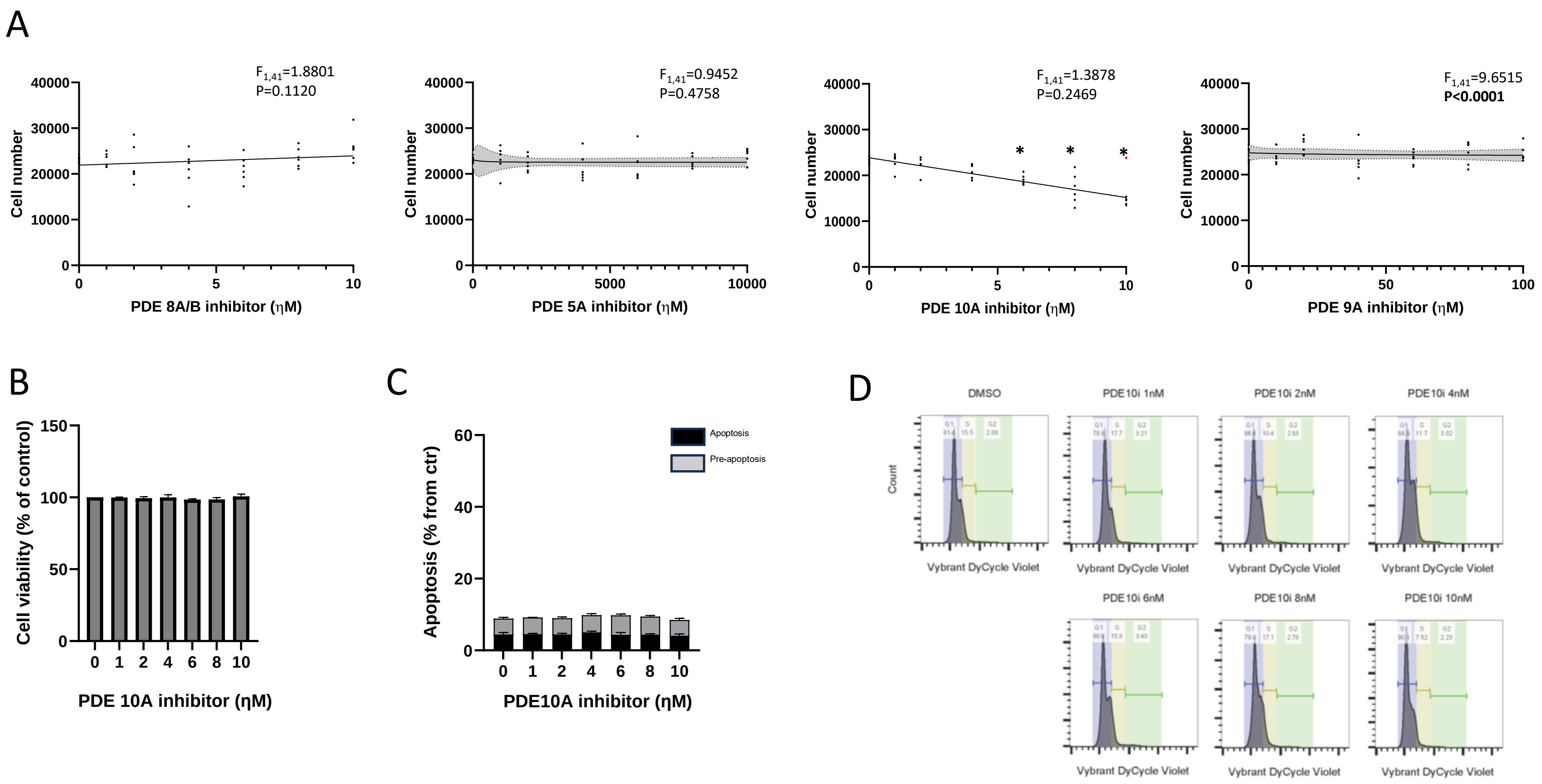

Supplemental Figure S4. PDEi effect on normal human bronchial epithelium cells. A. BEAS-2B in exponential phase of growth were exposed for 72h to PDE8i (0-10ηM), PDE5i (0-10000ηM), PDE9i (0-100ηM) or PDE10i (0-10ηM), followed by measurement of cell proliferation using WST-8 cell counting kit**.** BEAS-2B in exponential phase of growth were exposed for 72h to PDE10i (0-10ηM) and cell viability was measured assessed by **B.** Propidium Iodide (PI) viability assay and **C.** Annexin V-FITC / 7-ADD apoptosis assay. **D.** BEAS-2B in exponential phase of growth were exposed for 24h to PDE10i (0-10ηM) and cell proliferation was measured by flow cytometry using DyeCycle Violet Stain assay. Data presented as cell number, N=6. * Indicate significant differences from untreated respective control (P<0.05).B.

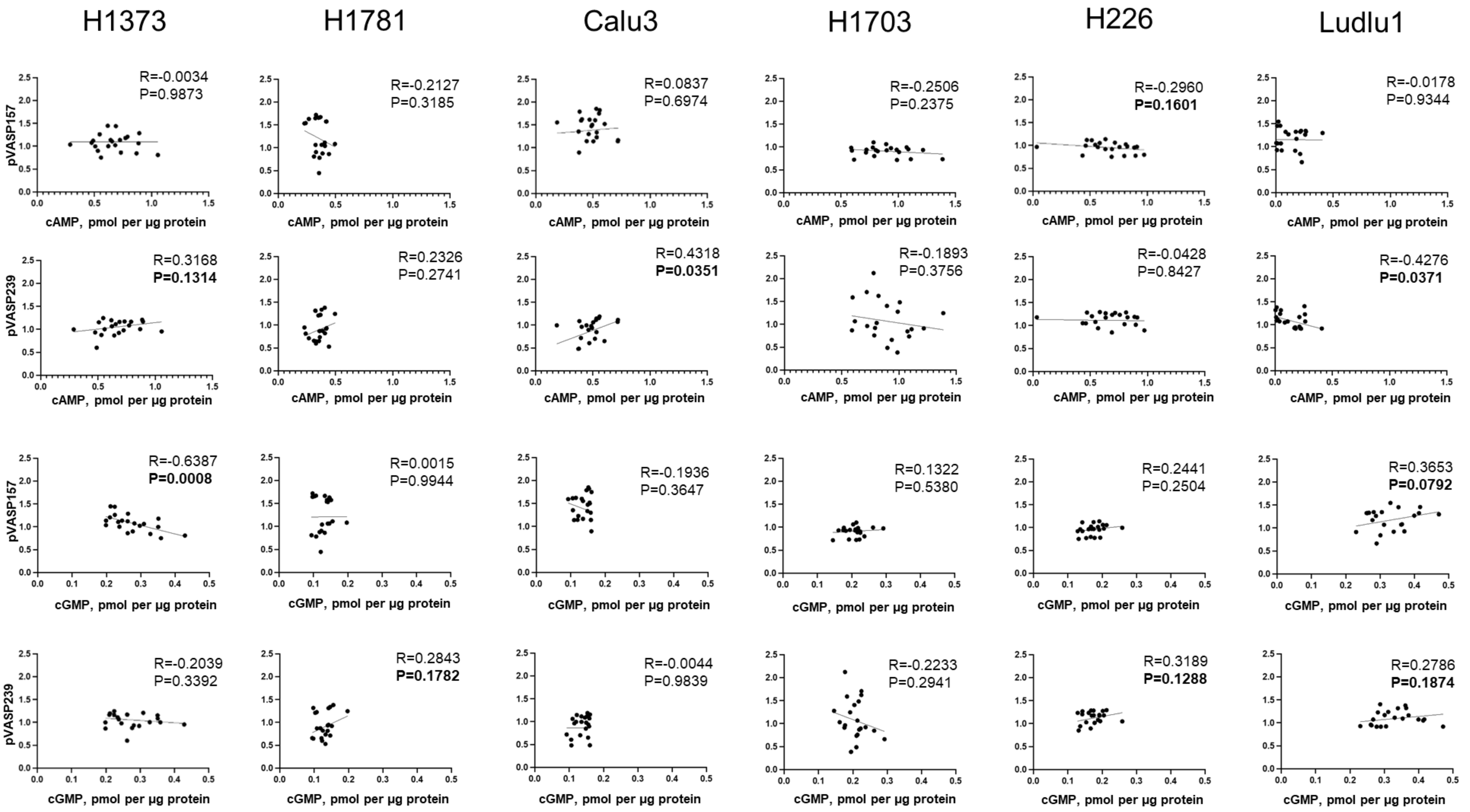

Supplemental Figure S5. Correlation between cyclic nucleotide concentration and protein kinases A or G activation. Correlation between changes in intracellular cyclic nucleotides (cAMP or cGMP) due to PDEi exposure and PKA and PKG activation (pVASP157 and pVASP239, respectively). Data from four PDEi were combined for each NSCLC cell lines. R-correlation, significantly different P value in bold, P<0.2.

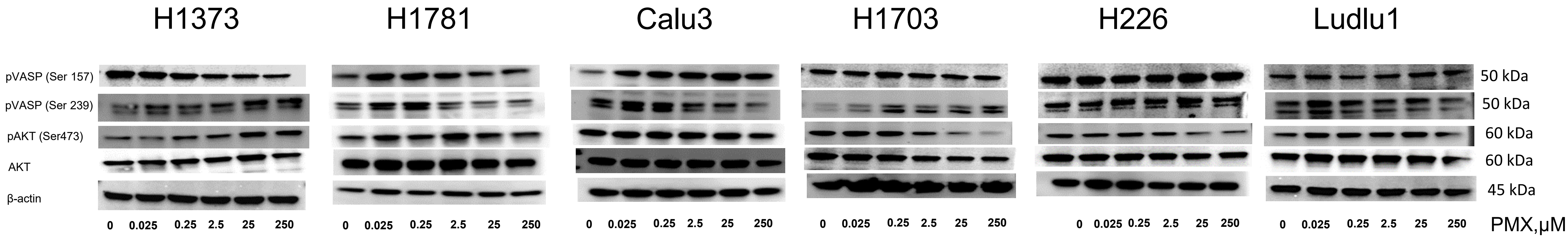

Supplemental Figure S6. PMX effect on PKA/PKG signaling in NSCLC cell lines. NSCLC cell lines (H1373, H1781, Calu3, H1703, H226 and Ludlu1) in exponential phase of growth were exposed for 72h to PMX (0, 0.025µM, 0.25µM, 2.5µM, 25µM and 250µM). Indirect PKA and PKG activity, as site-specific phosphorylation of vasodilator-stimulated phosphoprotein, PKA (pVASP157) and PKG (pVASP239), phospho-AKT (pAKT (Ser473)) and AKT determined by Western blotting. β-actin was used as the loading control.

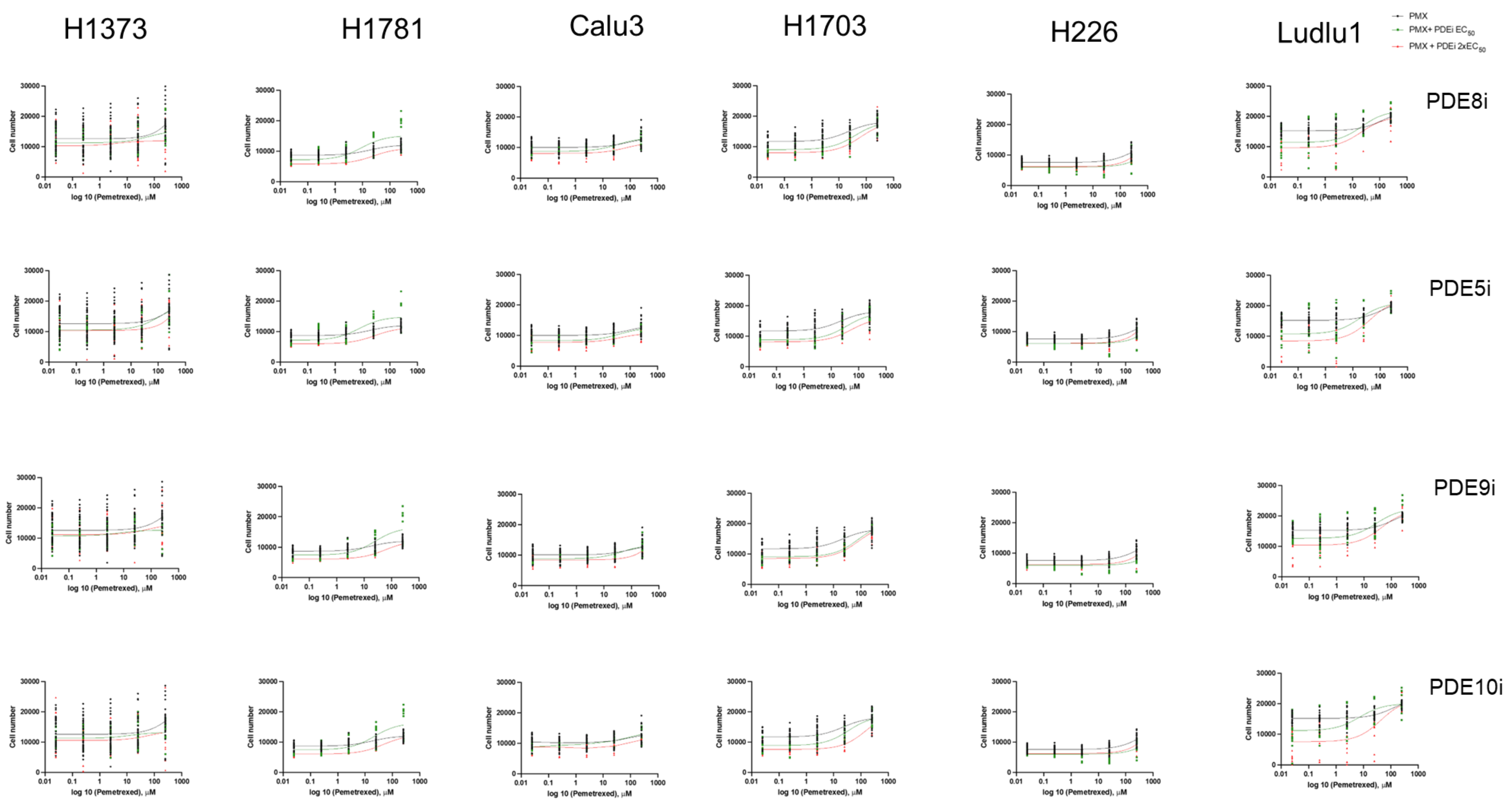

Supplemental Figure S7. Combination treatment of PMX and EC50 or 2xEC50 PDEi suppresses growth of some NSCLC cell lines. NSCLC cell lines (H1373, H1781, Calu3, H1703, H226 and Ludlu1) in exponential phase of growth were exposed for 72h to EC50 or 2x EC50 PDE8i, PDE5i, PDE9i or PDE10i in combination with PMX (0, 0.025µM, 0.25µM, 2.5µM, 25µM and 250µM), followed by measurement of cell proliferation using WST-8 cell counting kit. Data presented as cell number, N=6;9.

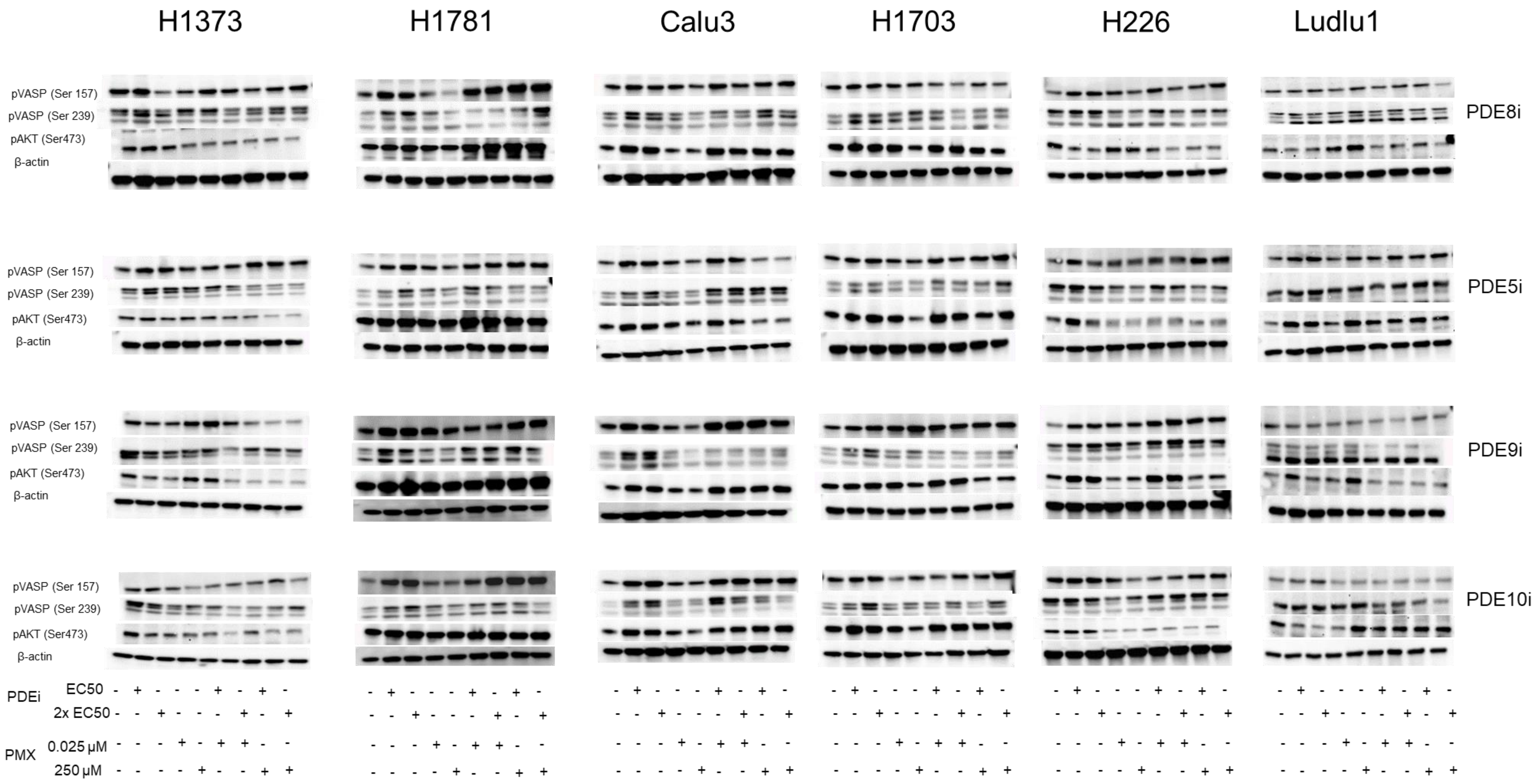

Supplemental Figure S8. Combination treatment of PMX and EC50 or 2xEC50 PDEi effect on PKA/PKG signaling in NSCLC. NSCLC cell lines (H1373, H1781, Calu3, H1703, H226 and Ludlu1) in exponential phase of growth were exposed for 72h to EC50 or 2x EC50 PDE8i, PDE5i, PDE9i or PDE10i in combination with PMX (0, 0.025 μM or 250 μM). Indirect PKA and PKG activity, as site-specific phosphorylation of vasodilator-stimulated phosphoprotein, PKA (pVASP157) and PKG (pVASP239), phospho-AKT (pAKT (Ser473) determined by Western blotting. β-actin was used as the loading control.

Supplemental Table S1. Presence of mutations in part of purine metabolism pathway in NSCLC. Cell lines without mutation highlighted in green

Website	Name	Tissue	Disease	cellular subtype	Age	Gender	Ethnicity	Smoking information	Genomic data		
ATCC	NCI-H1781 [H1781] (ATCC® CRL-5894™)	lung; derived from metastatic site: pleural effusion	stage 3B,adenocarcinoma; bronchoalveolar carcinoma	adenocarinoma	66	female	Caucasian	yes	Cosmic	CCLE	
ATCC	NCI-H1373 [H1373] (ATCC® CRL-5866™)	lung	stage 3A, adenocarcinoma	adenocarinoma	56	male	black	yes		CCLE	
ATCC	Calu-3 (ATCC® HTB-55™)	lung adenocarcinoma; derived from metastatic site: pleural effusion	adenocarcinoma	adenocarinoma	25	male	caucasian	n/a	Cosmic	CCLE	
ATCC	NCI-H1975 [H-1975, H1975] (ATCC® CRL-5908™	lung	adenocarcinoma; non-small cell lung cancer	adenocarinoma		female		no	Cosmic	CCLE	
ATCC	NCI-H1793 [H1793] (ATCC® CRL-5896™)	lung	adenocarcinoma; non-small cell lung cancer	adenocarinoma	52	female		no	Cosmic	CCLE	
Sigma	COR-L105 (ECACC 92031918)	lung	adenocarcinoma	adenocarinoma		male	Caucasian		Cosmic		
Sigma	LC-2/ad (ECACC 94072247)	lung	lung adenocarcinoma	adenocarinoma	51	female	japanese		Cosmic		
Sigma	PC-9 (ECACC 90071810)	lung	adenocarcinoma	adenocarinoma	45	male	japanese	no	Cosmic		
ATCC	NCI-H1568 [H1568] (ATCC® CRL-5876™)	lung; derived from metastatic site: lymph node	adenocarcinoma; non-small cell lung cancer	adenocarinoma	48	female	caucasian	yes	Cosmic	CCLE	
ATCC	NCI-H1563 [H1563] (ATCC® CRL-5875™)	lung	adenocarcinoma; non-small cell lung cancer	adenocarinoma		male		no	Cosmic	CCLE	
ATCC	NCI-H2342 [H2342] (ATCC® CRL-5941™)	lung	stage 3A, adenocarcinoma; non-small cell lung cancer	adenocarinoma	55	male	caucasian	no	Cosmic	CCLE	
ATCC	NCI-H2126 [H2126] (ATCC® CCL-256™)	lung; derived from metastatic site: pleural effusion	adenocarcinoma; non-small cell lung cancer	adenocarinoma	65	male	Caucasian	N/A	Cosmic	CCLE	
ATCC	NCI-H522 [H522] (ATCC® CRL-5810™)	lung	stage 2,adenocarcinoma; non-small cell lung cancer	adenocarinoma	58	male	caucasian	yes	Cosmic	CCLE	
ATCC	NCI-H1623 [H1623] (ATCC® CRL-5881™)	lung; derived from metastatic site: lymph node	stage 3B, adenocarcinoma; non-small cell lung cancer	adenocarinoma	58	male	Caucasian	yes	Cosmic	CCLE	
ATCC	NCI-H838 [H838] (ATCC® CRL-5844™)	lung; derived from metastatic site: lymph node	3B, adenocarcinoma; non-small cell lung cancer	adenocarinoma	59	male	Caucasian	yes	Cosmic	CCLE	
ATCC	NCI-H1693 [H1693] (ATCC® CRL-5887™)	lung; derived from metastatic site: lymph node	stage 3B, adenocarcinoma; non-small cell lung cancer	adenocarinoma	55	female	Caucasian	yes	Cosmic	CCLE	
ATCC	NCI-H2030 [H2030] (ATCC® CRL-5914™)	lung; derived from metastatic site: lymph node	adenocarcinoma; non-small cell lung cancer	adenocarinoma		male		no	Cosmic	CCLE	
ATCC	NCI-H2073 [H2073] (ATCC® CRL-5918™)	lung	stage 3A, adenocarcinoma; non-small cell lung cancer	adenocarinoma	47	female	Caucasian	yes		CCLE	
ATCC	NCI-H2087 [H2087] (ATCC® CRL-5922™)	lung; derived from metastatic site: lymph node	stage 1, adenocarcinoma; non-small cell lung cancer	adenocarinoma	69	male	Caucasian	yes	Cosmic	CCLE	
ATCC	NCI-H2405 [H2405] (ATCC® CRL-5944™)	lung	stage 4, adenocarcinoma; non-small cell lung cancer	adenocarinoma	47	male	Caucasian	yes	Cosmic	CCLE	
ATCC	NCI-H1435 [H1435] (ATCC® CRL-5870™)	lung	adenocarcinoma; non-small cell lung cancer	adenocarinoma	35	female		no	Cosmic	CCLE	
ATCC	NCI-H1651 [H1651] (ATCC® CRL-5884™)	lung	adenocarcinoma; non-small cell lung cancer	adenocarinoma	71	male		no	Cosmic	CCLE	
ATCC	NCI-H1734 [H-1734, H1734] (ATCC® CRL-5891™	lung	adenocarcinoma; non-small cell lung cancer	adenocarinoma	56	female		no	Cosmic	CCLE	
ATCC	NCI-H2023 [H2023] (ATCC® CRL-5912™)	lung	stage 3A, adenocarcinoma; non-small cell lung cancer	adenocarinoma	26	male	Caucasian	no	Cosmic	CCLE	
ATCC	NCI-H1993 [H1993] (ATCC® CRL-5909™)	lung; derived from metastatic site: lymph node	stage 3A, adenocarcinoma; non-small cell lung cancer	adenocarinoma	47	female	Caucasian	yes	Cosmic		
ATCC	NCI-H2228 [H2228] (ATCC® CRL-5935™)	lung	adenocarcinoma; non-small cell lung cancer	adenocarinoma		female		no	Cosmic	CCLE	
ATCC	NCI-H2291 [H2291] (ATCC® CRL-5939™)	lung; from lymph node, a metastatic site.	adenocarcinoma; non-small cell lung cancer	adenocarinoma		male		no	Cosmic	CCLE	
ATCC	NCI-H1437 [H1437] (ATCC® CRL-5872™)	lung; derived from metastatic site: pleural effusion	stage 1, adenocarcinoma; non-small cell lung cancer	adenocarinoma	60	male	Caucasian	yes	Cosmic	CCLE	
ATCC	NCI-H2122 [H2122] (ATCC® CRL-5985™)	lung; derived from metastatic site: pleural effusion	stage 4, adenocarcinoma; non-small cell lung cancer	adenocarinoma	46	female	Caucasian	yes	Cosmic	CCLE	
ATCC	NCI-H1944 [H1944] (ATCC® CRL-5907™)	lung; derived from metastatic site: soft tissue	stage 3B, adenocarcinoma; non-small cell lung cancer	adenocarinoma	62	female	Caucasian	yes	Cosmic	CCLE	
ATCC	NCI-H2347 [H2347] (ATCC® CRL-5942™)	lung	stage 1, adenocarcinoma; non-small cell lung cancer	adenocarinoma	54	female	Caucasian	no	Cosmic	CCLE	
ATCC	NCI-H1838 [H1838] (ATCC® CRL-5899™)	lung	adenocarcinoma; non-small cell lung cancer	adenocarinoma		female		no	Cosmic	CCLE	
ATCC	NCI-H23 [H23] (ATCC® CRL-5800™)	lung	adenocarcinoma; non-small cell lung cancer	adenocarinoma	51	male	black		Cosmic	CCLE	NCI60
ATCC	NCI-H1755 [H1755] (ATCC® CRL-5892™)	lung; derived from metastatic site: liver	stage 4, adenocarcinoma; non-small cell lung cancer	adenocarinoma	65	female	Caucasian	yes	Cosmic	CCLE	
ATCC	A549 (ATCC® CCL-185™)	lung	adenocarcinoma; non-small cell lung cancer	adenocarinoma	58	male	Caucasian		Cosmic		
ATCC	SK-LU-1 (ATCC® HTB-57™)	lung	lung adenocarcinoma (grade III)	adenocarinoma	60	female	Caucasian		Cosmic	CCLE	
ATCC	NCI-H1648 [H1648] (ATCC® CRL-5882™)	lung; derived from metastatic site: lymph node	stage 3A,adenocarcinoma	adenocarinoma	39	male	black	yes	Cosmic	CCLE	
ATCC	NCI-H810 [H810] (ATCC® CRL-5816)	lung	stage 2,carcinoma, non-small cell lung cancer	adenocarcinoma	51	male	black	N/A	Cosmic	CCLE	
ATCC	NCI-H727 [H727] (ATCC® CRL-5815™)	lung, bronchus	non-small cell carcinoma	adenocarcinoma	65	female	Caucasian		Cosmic	CCLE	
ATCC	NCI-H1573 [H1573] (ATCC® CRL-5877™)	lung; derived from metastatic site: soft tissue	adenocarcinoma (stage 4)	adenocarcinoma	35	female	Caucasian	yes		CCLE	
ATCC	NCI-H1703 [H1703] (ATCC® CRL-5889™)	lung	stage 1 lung squamous cell carcinoma	squamous cell carcinoma	54	male	caucasian	yes	Cosmic	CCLE	
Sigma	LUDLU-1 (ECACC 92012463)	lung	squamous cell carcinoma	squamous cell carcinoma	72	male	Caucasian	N/A		CCLE	
ATCC	NCI-H226 [H226] (ATCC® CRL-5826™)	lung; derived from metastatic site: pleural effusion	squamous cell carcinoma	squamous cell carcinoma		male			Cosmic	CCLE	NCI60
ATCC	NCI-H1869 [H1869] (ATCC® CRL-5900™)	lung	stage 4, non-small cell lung cancer	squamous cell carcinoma	58	male	Caucasian	N/A	Cosmic	CCLE	
ATCC	SK-MES-1 (ATCC® HTB-58™)	lung; Derived from Metastatic Site: pleural effusion	squamous cell carcinoma	squamous cell carcinoma	65	male	Caucasian	N/A	Cosmic	CCLE	
ATCC	NCI-H2170 [H2170] (ATCC® CRL-5928™)	lung	squamous cell carcinoma	squamous cell carcinoma		male		no	Cosmic	CCLE	
ATCC	NCI-H520 [H520] (ATCC® HTB-182™)	lung	squamous cell carcinoma	squamous cell carcinoma		male		N/A	Cosmic	CCLE	
ATCC	SW 900 [SW-900, SW900] (ATCC® HTB-59™)	lung	grade IV,squamous cell carcinoma	squamous cell carcinoma	53	male	Caucasian	N/A		CCLE	

Name	TP53	KRAS	PIK3CA	NRAS	PTEN	EGFR	ALK	HER2	METex14	BRAF	ROS1	RET	NTRK
NCI-H1781 [H1781] (ATCC® CRL-5894™)	x				x								x
NCI-H1373 [H1373] (ATCC® CRL-5866™)	x	x					x						
Calu-3 (ATCC® HTB-55™)	x												
NCI-H1975 [H-1975, H1975] (ATCC® CRL-5908™	x		x			x							
NCI-H1793 [H1793] (ATCC® CRL-5896™)	x					x	x						
COR-L105 (ECACC 92031918)													
LC-2/ad (ECACC 94072247)	x												
PC-9 (ECACC 90071810)	x					x	x						
NCI-H1568 [H1568] (ATCC® CRL-5876™)	x					x							
NCI-H1563 [H1563] (ATCC® CRL-5875™)			x									x	x
NCI-H2342 [H2342] (ATCC® CRL-5941™)	x												x
NCI-H2126 [H2126] (ATCC® CCL-256™)	x												x
NCI-H522 [H522] (ATCC® CRL-5810™)	x											x	
NCI-H1623 [H1623] (ATCC® CRL-5881™)	x												
NCI-H838 [H838] (ATCC® CRL-5844™)	x												
NCI-H1693 [H1693] (ATCC® CRL-5887™)							x						
NCI-H2030 [H2030] (ATCC® CRL-5914™)	x	x											x
NCI-H2073 [H2073] (ATCC® CRL-5918™)	x												
NCI-H2087 [H2087] (ATCC® CRL-5922™)	x			x			x			x			x
NCI-H2405 [H2405] (ATCC® CRL-5944™)	x									x			
NCI-H1435 [H1435] (ATCC® CRL-5870™)	x						x						x
NCI-H1651 [H1651] (ATCC® CRL-5884™)	x						x			x			
NCI-H1734 [H-1734, H1734] (ATCC® CRL-5891™	x	x											x
NCI-H2023 [H2023] (ATCC® CRL-5912™)	x												
NCI-H1993 [H1993] (ATCC® CRL-5909™)	x										x		
NCI-H2228 [H2228] (ATCC® CRL-5935™)	x											x	
NCI-H2291 [H2291] (ATCC® CRL-5939™)	x	x				x							x
NCI-H1437 [H1437] (ATCC® CRL-5872™)	x												x
NCI-H2122 [H2122] (ATCC® CRL-5985™)	x	x											
NCI-H1944 [H1944] (ATCC® CRL-5907™)		x											x
NCI-H2347 [H2347] (ATCC® CRL-5942™)		x		x								x	
NCI-H1838 [H1838] (ATCC® CRL-5899™)	x												
NCI-H23 [H23] (ATCC® CRL-5800™)	x	x										x	
NCI-H1755 [H1755] (ATCC® CRL-5892™)	x						x			x			
A549 (ATCC® CCL-185™)		x											
SK-LU-1 (ATCC® HTB-57™)	x	x											
NCI-H1648 [H1648] (ATCC® CRL-5882™)	x												
NCI-H810 [H810] (ATCC® CRL-5816)	x												x
NCI-H727 [H727] (ATCC® CRL-5815™)	x	x											x
NCI-H1573 [H1573] (ATCC® CRL-5877™)	x	x										x	x
NCI-H1703 [H1703] (ATCC® CRL-5889™)													
LUDLU-1 (ECACC 92012463)	x												
NCI-H226 [H226] (ATCC® CRL-5826™)													
NCI-H1869 [H1869] (ATCC® CRL-5900™)	x												x
SK-MES-1 (ATCC® HTB-58™)	x											x	
NCI-H2170 [H2170] (ATCC® CRL-5928™)	x												
NCI-H520 [H520] (ATCC® HTB-182™)	x												x
SW 900 [SW-900, SW900] (ATCC® HTB-59™)	x	x					x						x

Name	ADCY1	ADCY10	ADCY2	ADCY3	ADCY4	ADCY5	ADCY6	ADCY7	ADCY8	ADCY9	GUCY1A1	GUCY1A2	GUCY1B1	GUCY2C	GUCY2D	GUCY2F	NPR1	NPR2
NCI-H1781 [H1781] (ATCC® CRL-5894™)																		
NCI-H1373 [H1373] (ATCC® CRL-5866™)																		
Calu-3 (ATCC® HTB-55™)																		
NCI-H1975 [H-1975, H1975] (ATCC® CRL-5908™																		
NCI-H1793 [H1793] (ATCC® CRL-5896™)																		
COR-L105 (ECACC 92031918)																		
LC-2/ad (ECACC 94072247)																		
PC-9 (ECACC 90071810)			x		x			x		x								
NCI-H1568 [H1568] (ATCC® CRL-5876™)																		x
NCI-H1563 [H1563] (ATCC® CRL-5875™)																		
NCI-H2342 [H2342] (ATCC® CRL-5941™)			x						x			x						
NCI-H2126 [H2126] (ATCC® CCL-256™)												x						
NCI-H522 [H522] (ATCC® CRL-5810™)														x				
NCI-H1623 [H1623] (ATCC® CRL-5881™)	x													x				
NCI-H838 [H838] (ATCC® CRL-5844™)												x						
NCI-H1693 [H1693] (ATCC® CRL-5887™)																		
NCI-H2030 [H2030] (ATCC® CRL-5914™)							x		x									
NCI-H2073 [H2073] (ATCC® CRL-5918™)												x						
NCI-H2087 [H2087] (ATCC® CRL-5922™)																		
NCI-H2405 [H2405] (ATCC® CRL-5944™)							x											
NCI-H1435 [H1435] (ATCC® CRL-5870™)							x											
NCI-H1651 [H1651] (ATCC® CRL-5884™)	x	x				x		x										
NCI-H1734 [H-1734, H1734] (ATCC® CRL-5891™															x			
NCI-H2023 [H2023] (ATCC® CRL-5912™)																		
NCI-H1993 [H1993] (ATCC® CRL-5909™)		x										x						
NCI-H2228 [H2228] (ATCC® CRL-5935™)																		
NCI-H2291 [H2291] (ATCC® CRL-5939™)												x		x				
NCI-H1437 [H1437] (ATCC® CRL-5872™)																		
NCI-H2122 [H2122] (ATCC® CRL-5985™)																		
NCI-H1944 [H1944] (ATCC® CRL-5907™)																		
NCI-H2347 [H2347] (ATCC® CRL-5942™)		x																
NCI-H1838 [H1838] (ATCC® CRL-5899™)													x					
NCI-H23 [H23] (ATCC® CRL-5800™)					x											x		
NCI-H1755 [H1755] (ATCC® CRL-5892™)																x		
A549 (ATCC® CCL-185™)																		
SK-LU-1 (ATCC® HTB-57™)																		
NCI-H1648 [H1648] (ATCC® CRL-5882™)												x						
NCI-H810 [H810] (ATCC® CRL-5816)												x						
NCI-H727 [H727] (ATCC® CRL-5815™)																x		
NCI-H1573 [H1573] (ATCC® CRL-5877™)		x										x						
NCI-H1703 [H1703] (ATCC® CRL-5889™)																		
LUDLU-1 (ECACC 92012463)																		
NCI-H226 [H226] (ATCC® CRL-5826™)																		
NCI-H1869 [H1869] (ATCC® CRL-5900™)																		
SK-MES-1 (ATCC® HTB-58™)																		
NCI-H2170 [H2170] (ATCC® CRL-5928™)											x							
NCI-H520 [H520] (ATCC® HTB-182™)								x	x									
SW 900 [SW-900, SW900] (ATCC® HTB-59™)			x															

Name	PDE1A	PDE1B	PDE1C	PDE2A	PDE3A	PDE3B	PDE4A	PDE4B	PDE4C	PDE4D	PDE5A	PDE6A	PDE6B	PDE6C	PDE6D	PDE6G	PDE6H	PDE7A	PDE7B	PDE8A	PDE8B	PDE9A	PDE10A	PDE11A
NCI-H1781 [H1781] (ATCC® CRL-5894™)																								
NCI-H1373 [H1373] (ATCC® CRL-5866™)																								
Calu-3 (ATCC® HTB-55™)																								
NCI-H1975 [H-1975, H1975] (ATCC® CRL-5908™																								
NCI-H1793 [H1793] (ATCC® CRL-5896™)																								
COR-L105 (ECACC 92031918)																								
LC-2/ad (ECACC 94072247)																								
PC-9 (ECACC 90071810)			x										x											
NCI-H1568 [H1568] (ATCC® CRL-5876™)					x																			
NCI-H1563 [H1563] (ATCC® CRL-5875™)																							x	
NCI-H2342 [H2342] (ATCC® CRL-5941™)				x																	x	x		
NCI-H2126 [H2126] (ATCC® CCL-256™)																								
NCI-H522 [H522] (ATCC® CRL-5810™)					x					x														
NCI-H1623 [H1623] (ATCC® CRL-5881™)												x												
NCI-H838 [H838] (ATCC® CRL-5844™)																					x	x		
NCI-H1693 [H1693] (ATCC® CRL-5887™)									x															
NCI-H2030 [H2030] (ATCC® CRL-5914™)																								
NCI-H2073 [H2073] (ATCC® CRL-5918™)																								
NCI-H2087 [H2087] (ATCC® CRL-5922™)												x												
NCI-H2405 [H2405] (ATCC® CRL-5944™)																								
NCI-H1435 [H1435] (ATCC® CRL-5870™)																								
NCI-H1651 [H1651] (ATCC® CRL-5884™)						x																		
NCI-H1734 [H-1734, H1734] (ATCC® CRL-5891™																								
NCI-H2023 [H2023] (ATCC® CRL-5912™)					x	x					x													
NCI-H1993 [H1993] (ATCC® CRL-5909™)																								
NCI-H2228 [H2228] (ATCC® CRL-5935™)																					x			
NCI-H2291 [H2291] (ATCC® CRL-5939™)										x														
NCI-H1437 [H1437] (ATCC® CRL-5872™)		x											x											
NCI-H2122 [H2122] (ATCC® CRL-5985™)																							x	
NCI-H1944 [H1944] (ATCC® CRL-5907™)															x									
NCI-H2347 [H2347] (ATCC® CRL-5942™)																								
NCI-H1838 [H1838] (ATCC® CRL-5899™)																								
NCI-H23 [H23] (ATCC® CRL-5800™)	x							x																
NCI-H1755 [H1755] (ATCC® CRL-5892™)			x								x													
A549 (ATCC® CCL-185™)										x														
SK-LU-1 (ATCC® HTB-57™)											x													
NCI-H1648 [H1648] (ATCC® CRL-5882™)						x																		
NCI-H810 [H810] (ATCC® CRL-5816)																								
NCI-H727 [H727] (ATCC® CRL-5815™)																								
NCI-H1573 [H1573] (ATCC® CRL-5877™)	x			x	x			x					x				x				x	x		x
NCI-H1703 [H1703] (ATCC® CRL-5889™)																								
LUDLU-1 (ECACC 92012463)																								
NCI-H226 [H226] (ATCC® CRL-5826™)																								
NCI-H1869 [H1869] (ATCC® CRL-5900™)																								
SK-MES-1 (ATCC® HTB-58™)																								x
NCI-H2170 [H2170] (ATCC® CRL-5928™)																								
NCI-H520 [H520] (ATCC® HTB-182™)																								
SW 900 [SW-900, SW900] (ATCC® HTB-59™)					x																			

Supplementary Table S2. ANOVA results of the effect of single agent PDEi or PMX on cell.
F-values are given with degree of freedom for the factor and the error in subscript.
Significant values(P<0.05) are highlighted in bold.

**Cell line**		**PDE9a**	**PDE10a**	**PDE8a**	**PDE5a**	**PMX**
**H1373**	F Ratio	$F_{1,29}=58.8880$	$F_{1,29}=9.3112$	$F_{1,29}=77.7511$	$F_{1,29}=103.5557$	$F_{1,34}=38.7517$
	Prob>F	**<0.0001**	**0.00049**	**<0.0001**	**<0.0001**	**<0.0001**
**H1781**	F Ratio	$F_{1,23}=32.071$	$F_{1,23}=35.9462$	$F_{1,2}3=35.7650$	$F_{1,23}=40.1771$	$F_{1,39}=7.7786$
	Prob>F	**<0.0001**	**<0.0001**	**<0.0001**	**<0.0001**	**0.0082**
**Calu3**	F Ratio	$F_{1,28}=93.3952$	$F_{1,29}=66.4658$	$F_{1,28}=71.4507$	$F_{1,29}=41.3458$	$F_{1,34}=38.7517$
	Prob>F	**<0.0001**	**<0.0001**	**<0.0001**	**<0.0001**	**<0.0001**
**H1703**	F Ratio	$F_{1,23}=60.7316$	$F_{1,23}=34.2756$	$F_{1,23}=40.3321$	$F_{1,23}=48.2803$	$F_{1,39}=1.8059$
	Prob>F	**<0.0001**	**<0.0001**	**<0.0001**	**<0.0001**	0.187
**H226**	F Ratio	$F_{1,23}=17.9207$	$F_{1,23}=17.9207$	$F_{1,23}=23.6596$	$F_{1,23}=15.0978$	$F_{1,47}=5.8707$
	Prob>F	**<0.0001**	**0.0003**	**<0.0001**	**0.0008**	**0.0194**
**Ludlu1**	F Ratio	$F_{1,29}=0.7633$	$F_{1,29}=0.3032$	$F_{1,29}=1.6210$	$F_{1,29}=1.4292$	$F_{1,34}=38.7517$
	Prob>F	0.3897	0.5863	0.2134	0.2419	**<0.0001**

Supplementary Table S3. Single agent PDEi or PMX effect on cell proliferation. EC50 calculated for each cell line in the presence of PDEi (PDE8i, PDE5i, PDE9i or PDE10i). P value<0.05 calculated for maximum cell growth in the presence of PDEi.

Treatment	Cell line	**H1373**	**H1781**	**Calu3**	**H1703**	**H226**	**Ludlu1**
	Cell type	AC	AC	AC	SCC	SCC	SCC
	Doubling time,h	60.25	102.11	77.17	59.31	133.35	44.48
	EC50, ηM	1.54	5.44	3.56	2.08	5.16	n/a
PDE8i	Max growth (Fold change)	+ 1.71	+ 1.65	+ 2.86	+ 1.51	+ 1.51	1.00
	P value	**<.0001**	**<.0001**	**<.0001**	**<.0001**	**0.0065**	0.2134
	EC50, ηM	2423	2000	1937	5548	1582	n/a
PDE5i	Max growth (Fold change)	+ 1.68	+ 1.71	+ 2.30	+ 1.51	+ 1.33	1.09
	P value	**<.0001**	**<.0001**	**<.0001**	**<.0001**	**0.0008**	0.2419
	EC50, ηM	31.5	64.0	57.1	40.2	14.6	n/a
PDE9i	Max growth (Fold change)	+ 1.74	+ 1.73	+ 2.80	+ 1.55	+ 1.43	1.19
	P value	**<.0001**	**<.0001**	**<.0001**	**<.0001**	**0.0003**	0.3897
	EC50, ηM	0.50	1.60	1.20	4.05	1.15	n/a
PDE10i	Max growth (Fold change)	+ 1.45	+ 1.57	+ 2.58	+ 1.49	+ 1.40	1.04
	P value	**0.0049**	**<.0001**	**<.0001**	**<.0001**	**<.0001**	0.5863
	EC50,μM	7405	18.32	95.92	21.84	399.8	130.3
PMX	Max growth (Fold change)	+ 1.35	+ 1.47	+ 1.32	+ 1.45	+ 1.36	+ 1.30
	P value	**<.0001**	**0.0082**	**<.0001**	0.187	**0.0194**	**<.0001**

Supplementary Table S4. Single agent PDEi effect on indirect PKA /PKG activity, as site specific phosphorylation of vasodilator-stimulated phosphoprotein, PKA(pVASP157) and PKG (pVASP239). Significant values(P<0.05) are highlighted in bold.

		H1373		H1781		Calu3		H1703		H226		Ludlu1	
PDEi		correlation	P value	correlation	P value	correlation	P value	correlation	P value	correlation	P value	correlation	P value
**8i**	pVASP157	-0.3926	0.4414	0.5321	0.2772	0.2075	0.6932	-0.2962	0.5687	0.6206	0.1886	-0.5628	0.2449
**8i**	pVASP239	**-0.8284**	**0.0416**	-0.4535	0.3664	-0.1871	0.7226	**-0.8871**	**0.0184**	0.2842	0.5852	-0.6280	0.1818
**5i**	pVASP157	0.2958	0.5692	**-0.8821**	**0.02**	0.3612	0.4817	0.1706	0.7466	-0.4566	0.3627	-0.3781	0.4599
**5i**	pVASP239	-0.2894	0.5781	0.3963	0.4367	0.4847	0.3299	-0.5313	0.2781	0.7622	0.0781	0.6886	0.1303
**9i**	pVASP157	**-0.9823**	**0.0005**	0.6684	0.1467	0.1894	0.7193	-0.0394	0.941	**0.9565**	**0.0028**	0.3590	0.4846
**9i**	pVASP239	0.2316	0.6588	0.7558	0.0822	0.2579	0.6217	-0.5078	0.3038	0.4707	0.3461	0.1520	0.7737
**10i**	pVASP157	-0.3995	0.4326	0.4609	0.3576	**0.8205**	**0.0454**	-0.2141	0.6838	0.4727	0.3438	0.2756	0.597
**10i**	pVASP239	**-0.8298**	**0.041**	0.7701	0.0732	0.6204	0.1888	-0.0567	0.9151	**0.9152**	**0.0105**	0.4754	0.3406

Supplementary Table S5. ANOVA results of the effect of combined treatment PDEi and PMX on apoptosis and cell growth. F-values are given with degree of freedom for the factor and the error in subscript. Significant values(P<0.05) are highlighted in bold.

**Cell line**		**Apoptosis**	**Cell growth**
**H1373**	F Ratio	$F_{4,67}$=1.7497	$F_{4,269}$=5.4109
	Prob>F	0.1503	**0.0003**
**H1781**	F Ratio	$F_{4,67}$=1.1906	$F_{4,215}$=118.0590
	Prob>F	0.3237	**<0.0001**
**Calu3**	F Ratio	$F_{4,67}$=6.2231	$F_{4,215}$=54.3258
	Prob>F	**0.0003**	**<0.0001**
**H1703**	F Ratio	$F_{4,67}$=6.6725	$F_{4,213}$=177.5958
	Prob>F	**0.0002**	**<0.0001**
**H226**	F Ratio	$F_{4,67}$=0.9924	$F_{4,214}$=26.1698
	Prob>F	0.4183	**<0.0001**
**Ludlu1**	F Ratio	$F_{4,67}$=5.7481	$F_{4,206}$=117.5843
	Prob>F	0.0005	**<0.0001**

Supplementary Table S6. Comparison of PDEi and pemetrexed concentrations between *in vitro* experiments and concentrations being used in clinic.

Drug	Max dose tested in vitro, ng/ml	Concentration, administration	PK: Max serum concentration, ng/ml
PDE8i (PF-04671536)	3.82	No clinical data available	
PDE9i (PF-04447943)	4.28	5 mg, oral	48.5-59.5 (1)
PDE5i (Sildenafil citrate)	10,000	50 mg, infusion<br>50 mg, oral	531 (2)<br>159
PDE10i (PF-2545920)	4.37	5 mg, oral	17.6-36.5 (3)
Pemetrexed	106,000	400-700 mg m^{-2}, infusion	89,100-131,000 (4)

Supplementary References

1. Charnigo RJ, Beidler D, Rybin D, Pittman DD, Tan B, Howard J, et al. PF-04447943, a Phosphodiesterase 9A Inhibitor, in Stable Sickle Cell Disease Patients: A Phase Ib Randomized, Placebo-Controlled Study. Clinical and Translational Science. 2019;12(2):180-8.
2. Nichols DJ, Muirhead GJ, Harness JA. Pharmacokinetics of sildenafil after single oral doses in healthy male subjects: absolute bioavailability, food effects and dose proportionality. British journal of clinical pharmacology. 2002;53 Suppl 1(Suppl 1):5s-12s.
3. Walling DP, Banerjee A, Dawra V, Boyer S, Schmidt CJ, DeMartinis N. Phosphodiesterase 10A Inhibitor Monotherapy Is Not an Effective Treatment of Acute Schizophrenia. Journal of Clinical Psychopharmacology. 2019;39(6):575-82.
4. Li KM, Rivory LP, Clarke SJ. Pemetrexed pharmacokinetics and pharmacodynamics in a phase I/II study of doublet chemotherapy with vinorelbine: implications for further optimization of pemetrexed schedules. British journal of cancer. 2007;97(8):1071-6.
